# From LC-MS/MS metabolomics profiling of *Kanchanara Guggulu* to molecular docking and dynamics simulation of quercetin pentaacetate with aldose reductase

**DOI:** 10.6026/97320630017911

**Published:** 2021-11-30

**Authors:** Santosh Kumar Behera, Prashant Kumar Modi, Gayathree Karthikkeyan, Sameera Krishna Pervaje, Ravishankar Pervaje, Rajesh Raju, Thottethodi Subrahmanya Keshava Prasad, Yashwanth Subbannayya

**Affiliations:** 1 Center for Systems Biology and Molecular Medicine, Yenepoya Research Centre, Yenepoya (Deemed to be University), Mangalore 575018, India; 2 Yenepoya Medical College, Yenepoya (Deemed to be University), Mangalore 575018, India;; 3 Sushrutha Ayurveda Hospital, Bolwar, Puttur 574201, India; #Current address: Centre of Molecular Inflammation Research (CEMIR), Department of Clinical and Molecular Medicine (IKOM), Norwegian University of Science and Technology, N-7491 Trondheim, Norway

**Keywords:** LC-MS/MS, phytochemicals, aldose reductase inhibitor, Systems Biology

## Abstract

Kanchanara Guggulu (KG) is an important traditional medicine that is prescribed by the Ayurveda physicians for the treatment of swellings in various organs such as the thyroid, and lymph nodes. High-resolution mass-spectrometry-based metabolomics found metabolites in KG. LC-MS/MS-based metabolomics analysis of KG identified 2,579 compounds including quercetin and kaempferol derivatives. The molecular docking and dynamics analysis of quercetin pentaacetate with aldose reductase is documented for further consideration in drug discovery.

## Background:

Ayurveda is a well-known codified traditional medicinal practice in India as a complementary and alternate therapy. One of the vital drug formulations in Ayurveda is Kanchanara Guggulu (KG) which is a mixture of several ingredients, including Bauhinia variegata (Kanchanara), Emblika officinalis (Amalaki), Terminalia chebula (Haritaki), Terminalia bellirica (Bibhitaki), Piper longum (Pippali), Zingiber officinale (Shunthi), and Piper Nigrum (Maricha) [1]. KG is prescribed by the physicians for treatment of lymphatic and thyroid swellings. B. variegata, the major constituent plant of KG has several other properties such as anti-cancer, anti-diabetic and anti-inflammatory [2, 3]. KG primarily consists of B. variegata, rich in phytochemicals such as terpenoids, phenolics, flavonoids, anthraquinones, saponins, tannins, and alkaloids [4]. Flavonoids (glucopyranoside derivatives of kaempferol, isorhamnetin, and hesperidin) and triterpenoids extracted from the B. variegata showed their effectiveness with anti-inflammatory activities. Although there are studies available for identifying and quantifying a selected set of metabolites in Kanchanara [5,6], global profiling of KG metabolites has not been carried out to date. Therefore, it is of interest to document data from LC-MS/MS based metabolomics profiling of Kanchanara Guggulu to molecular docking and dynamics analysis of Quercetin pentaacetate with aldose reductase.

## Materials & Methods:

### Material procurement: 

The current study has been approved by the Institutional Scientific Review Board (YRC-SRB/019/2018). The formulation (Lot No. 28) was procured from SDP Remedies and Research Centre, Puttur, Karnataka. The sample specimen was maintained at SDP Remedies and Research Centre with the identifier SDP/KG/001-2017. Sample preparation and LC-MS/MS-based metabolomic profiling KG sample was incubated for 1 min with the extraction solvent (methanol, acetonitrile, and water in 2:2:1 ratio) [7], followed by the sonication for 10 min. The samples were then centrifuged at 12,000 g for 15 min at 4°C. The resultant supernatant was collected and stored at -20°C until the LC-MS/MS analysis.

Liquid chromatography (LC) was used to extract the metabolites, followed by MS/MS analysis using QTRAP 6500 (AB SCIEX, USA). The metabolites were separated for 20 minutes in the LC method. Two solvents A (0.1% formic acid in MilliQ water) and B (90% acetonitrile), were used as the mobile phase with a flow rate of 0.3 mL/min. LC method was carried out with 2% B at t=0-1 min; 30% B at t=10 min; 60% B at t=11 min; 95% B at t=13-17 min and 2% B at t=17.2-20 min and allowed the elution of metabolites in these gradients. Information Dependent Acquisition (IDA) in low mass mode built using Enhanced Mass Spectra (EMS) to Enhanced Production (EPI) modes were used for MS data acquisition. The top five spectra from the EMS mode were used for analysis in the EPI-MS/MS mode, using high Collisionally Induced Dissociation (CID) energy. The data were acquired in positive (4500 V) and negative (-4500V) mode with a probe temperature of 450°C. The Declustering Potential (DP) and Collision Energy (CE) were set as 100V and 40V, respectively, for the compound parameter. The data acquisition was done in triplicates.

### Compound identification:

The raw files were processed and searched against the METLIN database through the XCMS server [8]. Further, the raw files were pre-processed using MZmine2 [9], and MS/MS features were generated in Mascot Generic Format (MGF). Next, the MS/MS search was performed in MS2Compound [10] by mapping to PlantCyc [11], KEGG secondary metabolites [12], and Phenol-Explorer [13] with 0.05 Da m/z tolerance for both precursor and fragment levels. Finally, compounds identified from both platforms were merged to generate a final list of compounds.

### Molecular docking:

Manual curation was undertaken to enlist the known metabolites in the Bauhinia species. All these metabolites were checked for their potential target proteins, which may have a significant role in many diseases such as cancer or diabetes. Quercetin pentaacetate was found to bind with aldose reductase from bindingDB analysis. Aldose reductase inhibitors are known to be effective against diabetes. Therefore, we performed a molecular docking study to analyze the interaction between these two molecules. A high-resolution crystal structure (at 0.66 Å) of Aldose reductase (PDB ID: 1US0) along with the cofactor NADP+ and the inhibitor IDD594 was considered for the study [14]. The molecular docking was performed using AutoDock 4.2 [15].

To check the efficiency of AutoDock, the binding pose of the known inhibitor was reconstructed prior to docking the selected molecule. The macromolecule was prepared in the WHAT IF server [16]. Water molecules were removed, and polar hydrogens were added to the macromolecule. A grid was generated with 56, 44, and 60 points in x, y, and z- dimensions, respectively, centered at 17.519, -6.308, 17.212 at x, y, and z coordinates. The grid box was generated considering the active site of the known structure. Docking was performed for 500 conformations using the Genetic algorithm. The same grid box and parameters were used for docking quercetin pentaacetate to aldose reductase. Both the dockings were performed, allowing rotation of all the rotatable bonds of the ligands.

### Molecular dynamics simulation:

The stability of quercetin pentaacetate and aldose reductase complex was analyzed using a molecular dynamics (MD) simulation study in GROningen Machine for Chemical Simulations (GROMACS) version 2019.6 computational package [17]. The MD simulation steps were adopted from a previous study [18]. Topology parameters of the ligand quercetin and the cofactor NADP were generated using Automated Topology Builder (ATB) [19]. The AMBER99SB-ILDN force field [20] was used along with a simple point charge (SPC) as the water model for generating the topology of the macromolecule. Energy minimization was performed for 50000 steps with the steepest descent minimization algorithm. A modified Berendsen thermostat algorithm (V-rescale) was used to regulate the temperature and pressure of the system. The system's temperature was maintained at a room temperature of 300K with 1 bar pressure for 100 ps. Finally, an MD simulation was performed for 50 ns for the protein-ligand-cofactor complex.

## Results and Discussion:

A total of 2,579 non-redundant compounds were identified from KG metabolomics data, including the prior-known compounds such as kaempferide 3-rhamnoside-7-(6''-succinylglucoside), luteolin 7-sulfate, and caffeoyl-CoA. The complete list of identified metabolites is provided in Zenodo (File name: Supplementary File S1.xlsx) [21]. In the current study, we also found previously identified metabolites of Bauhinia species. Some of them include glucoside derivatives of kaempferol such as kaempferol 3-O-[6-(4-coumaroyl)-beta-D-glucosyl-(1->2)-beta-D-glucosyl-(1->2)-beta-D-glucoside]; and kaempferide 3-rhamnoside-7-(6''-succinylglucoside); Quercetin derivatives such as quercetin 3,7,3'-tri-O-sulfate, and quercetin pentaacetate were identified in the current analysis. A list of previously known metabolites which are also identified in the current study is provided in Table 1(see PDF).

We performed a molecular docking followed by a molecular dynamics simulation study to show the anti-diabetic property of quercetin pentaacetate, a known compound in Bauhinia species. The known inhibitor of Aldose reductase was docked to the macromolecule to check the efficiency of AutoDock in reconstructing the protein-ligand binding pattern. IDD594 was found to form three hydrogen bonds with Aldose reductase at Tyr 48, His 110, and Trp 111 with -10.54 kJ/mol binding energy (Figure 1A - see PDF). Our results suggest AutoDock could generate three out of the four known hydrogen bonds of the same pair. This provides confidence to check the binding of quercetin pentaacetate with aldose reductase. Docking of quercetin pentaacetate with aldose reductase resulted in four hydrogen bonds (Trp 20, Tyr 48, His 110, and Leu 301) with -9.93 kJ/mol binding energy (Figure 1B - see PDF). A molecular dynamics simulation study supported the docking study. The system's potential energy was found to be -665000 kJ/mol throughout the 50ns stable throughout the 30ns simulation time frame (Figure 1C - see PDF). The RMSD of the complex was found to be stable (<1.5 Å) after a 10ns period (Figure 1D - see PDF). The root mean square fluctuation (RMSF) of most of the residues was found to be <1.5 Å suggesting a stable complex (Figure 1E - see PDF). A steady value of Rg (radius of gyration) was found to be maintained throughout 50ns (Figure 1F - see PDF). The RMSD and RMSF value also suggested the stable conformation, with an average of one hydrogen bond. This confirms quercetin pentaacetate as a potential aldose reductase inhibitor. Aldose reductase inhibitors are known to prevent diabetes by controlling the polyol pathway [22,23]. Studies also suggest Bauhinia species possess anti-diabetic properties [24,25]. Further, quercetin is known as one of the aldose inhibitors [26], indicating that this quercetin derivative might potentially serve as an aldose inhibitor.

## Conclusion:

We document data from LC-MS/MS based metabolomics profiling of Kanchanara Guggulu to molecular docking and dynamics analysis of Quercetin pentaacetate with aldose reductase.

## Author Disclosure statement:

The authors declare that they have no competing financial interests.

## References

[1] Dhiman K (2014). Ayu..

[4] Mishra A (2013). Biomed Res Int.

[5] Rao YK (2008). Phytother Res.

[6] Yadava RN, Reddy VM (2003). Nat Prod Res.

[7] Lau SK (2015). Emerg Microbes Infect.

[8] Domingo-Almenara X, Siuzdak G (2020). Methods Mol Biol.

[9] Pluskal T (2010). BMC Bioinformatics.

[10] Behera SK (2021). OMICS.

[11] Schlapfer P (2017). Plant Physiol.

[12] Kanehisa M (2019). Protein Sci.

[13] Rothwell JA (2012). Database (Oxford).

[14] Howard EI (2004). Proteins.

[15] Morris GM (2009). J Comput Chem.

[17] Pronk S (2013). Bioinformatics.

[18] Arya H, Coumar MS (2014). J Mol Model.

[19] Koziara KB (2014). J Comput Aided Mol Des.

[20] Lindorff-Larsen K (2010). Proteins.

[22] Turkes C (2019). Appl Biochem Biotechnol.

[23] Hu X (2014). PLoS One.

[24] Cechinel Filho V (2009). Phytother Res.

[25] Kulkarni YA, Garud MS (2016). Biomed Pharmacother.

[26] Reddy TN (2014). Eur J Med Chem.

[28] dos Santos AE (2014). Parasit Vectors.

[29] Huang J (2021). Chemical Biology & Drug Design.

[30] Braca A (2001). J Nat Prod.

[31] Lopes BRP (2020). Virus Res.

[32] Kaewamatawong R (2008). J Nat Med.

[33] de Sousa E (2004). J Nat Prod.

[34] Pinto LS (2008). J Biosci.

